# Ultra‐processed food supply in hospitals: A cross‐sectional analysis of Australian hospitals using cook fresh foodservices

**DOI:** 10.1111/1747-0080.70083

**Published:** 2026-06-04

**Authors:** Priscila Machado, Nathan Cook, Mark Lawrence, Mike Forrester, Rebecca Patrick, Judi Porter

**Affiliations:** ^1^ Institute for Physical Activity and Nutrition, School of Exercise and Nutrition Sciences Deakin University Geelong Victoria Australia; ^2^ Faculty of Health, Medicine and Behavioural Sciences, School of Human Movement and Nutrition Sciences University of Queensland Brisbane Queensland Australia; ^3^ Institute for Health Transformation, School of Health and Social Development, Faculty of Health Deakin University Geelong Victoria Australia; ^4^ School of Population and Global Health The University of Melbourne Melbourne Victoria Australia

**Keywords:** food processing, food supply, healthcare, hospital, institutional foodservice

## Abstract

**Aim:**

Ultra‐processed foods have been associated with a range of adverse health and environmental impacts, yet their supply in health‐promoting settings like hospitals remains largely unexamined. This study aimed to assess and quantify the proportion of ultra‐processed foods utilised in a sample of Australian hospitals using cook fresh foodservice systems (i.e., most menu items are prepared/cooked just prior to service), and to determine types of ultra‐processed foods available in each of the menu categories.

**Methods:**

Cross‐sectional data on the foods and drinks available to inpatients in Australian hospitals were collected in October and November 2023. Trained hospital dietitians recorded the standard inpatient menu (Full ward diet), short‐order menu items, and oral nutrition support products for paediatric and adult patients over seven consecutive days. Australian food composition databases were used to estimate the energy content of each item. The number of items and energy contribution of ultra‐processed foods was estimated by meal (e.g., breakfast, lunch, dinner) and food category (e.g., cereals, spreads, dairy).

**Results:**

Six hospitals using predominantly or cook fresh only foodservice systems were included, offering 100–1200 meals per day. Ultra‐processed foods contributed 51% of total energy provided, with the highest proportions observed at breakfast and afternoon tea. Bread, dessert, snack, spread/sauce, and drinks had over two‐thirds of items classified as UPFs.

**Conclusions:**

Findings suggest a concerning large contribution of ultra‐processed foods in Australian hospital cook fresh foodservice systems. These findings have implications for interventions aimed at promoting both public and planetary health in institutional settings.

## 1 INTRODUCTION

Ultra‐processed foods (UPFs) are becoming dominant in food systems and human diets, driving significant negative impacts on both public and planetary health.^1^ UPFs are formulations of ingredients, mostly of exclusive industrial use (e.g., hydrogenated oils, protein isolates, maltodextrin, flavours, colours, thickeners, emulsifiers), but few whole foods.^2^ UPF sales are increasing nearly everywhere,^1^ and these foods are the main source of dietary energy in many high‐income countries (e.g., Canada, UK, US).^3^ In Australia, UPFs contribute to 56% of total energy purchased by Australian households^4^ and 42% of total daily energy intake of Australians,^5^ with highest intakes observed among children, adolescents and young adults^6^ and the most disadvantaged.^7^

Dietary exposure to UPFs is associated with over 30 adverse health outcomes,^8^ such as type‐2 diabetes, depression, heart diseases, and premature death, including in Australia.^9^, ^10^, ^11^, ^12^ These adverse health impacts can be explained by the combination of the poor nutritional quality of diets based on UPFs,^5^, ^7^, ^13^ the presence of additives (e.g., emulsifiers, artificial sweeteners) and contaminants (e.g., advanced glycation end products, phthalates from plastic packaging) that are endocrine disruptors and the displacement of minimally processed foods.^14^ UPFs are often ready‐to‐eat, palatable, affordable, heavily promoted, and conveniently packaged products,^3^ designed to be overconsumed and easily ingested, leading to excess energy intake.^15^

The increased production, supply, and consumption of UPFs harm the environment by generating excessive packaging waste, chemical pollution, and greenhouse gas emissions.^16^ They require substantial energy, water, and land for the cultivation and processing of commodity ingredients (e.g., corn, soy, sugarcane) that form the basis of many UPFs—leading to deforestation and associated species extinction (biodiversity loss).^16^, ^17^ The dominance of food systems by powerful UPF corporations and their affiliates undermines small‐ and medium‐sized food businesses, which struggle to compete against large‐scale manufacturers with greater capital and market influence.^18^, ^19^

The UPF concept is now widely recognised as relevant for informing research, policy, and practice, as evidenced by the extensive growth in the body of evidence,^8^, ^20^ its inclusion into an increasing number of national dietary guidelines,^21^ and policy reports from United Nations bodies such as the World Health Organization and the Food and Agriculture Organization.^20^, ^22^ The role of policies and interventions in improving institutional settings, such as hospitals, has been increasingly recognised.^23^, ^24^ Hospitals have a responsibility to promote public and planetary health,^23^, ^25^ as they exert influence at the individual (e.g., short‐term dietary pattern and nutritional adequacy), local (e.g., as a model for community health, impacting local suppliers), and systems (e.g., high resource use, food and packaging waste) levels.^23^, ^25^, ^26^, ^27^ Hospitals and healthcare settings are well‐positioned—and expected—to lead by example in implementing low‐UPF food provision models. However, providing healthy and sustainable foods in these settings is complex, shaped by organisational practices and the need to meet diverse dietary requirements outlined in Nutrition Standards.^28^, ^29^, ^30^

Research on UPF supply in hospitals is emerging (e.g., Brazil and Greece),^31^, ^32^ but none has been conducted in Australia. To address this gap in the literature, and provide much needed evidence to guide practice and policy on hospitals food provision, this study aimed to assess and quantify the proportion of UPFs utilised in a sample of Australian hospitals using cook fresh food service systems, and to determine key types of UPFs available in each of the menu categories.

## 2 METHODS

This study used a cross‐sectional design to analyse the UPF supply in Australian hospitals. Hospitals were eligible if they only or predominantly used a cook fresh foodservice system (i.e., the majority of menu items are prepared/cooked just prior to service). Including hospitals with cook fresh systems allowed for a more accurate assessment of the utilisation of UPFs, as ingredients and recipe components were prepared on‐site and readily accessible. Hospitals were ineligible if their foodservice system predominantly relied on food produced in advance of the meal (i.e., hospitals using cook chill or cook freeze models).

Mapping of eligible hospitals was conducted by the research team with a convenience sample of Australian hospitals invited to participate in the study in July–August 2023. Hospitals were approached by the research team to verify their eligibility, then invited to participate. Following the receipt of the hospital's agreement letter and signed consent form, one dietitian from each hospital received training from the research team. Training occurred during a 90‐min online training session via Zoom regarding the collection of the standard inpatient menu (full ward diet), short‐order menu items, and oral nutrition support products for paediatric and adult patients over seven consecutive days. Menus were designed for each hospital for inpatients following a full ward diet. Enteral, parenteral and other therapeutically required menus (e.g., soft, liquid, texture modified, coeliac diets) were excluded. Foods purchased through vending machines or retail food outlets located in the hospitals and any foods brought in from family that were not part of the inpatient menu were excluded. A recording of the online training and data collection manual was provided at the time of data collection (Data S1, Supporting Information). Data collection was undertaken in October–November 2023.

Forms for data collection were created by the research team (Data S2). Trained dietitians inserted into Microsoft Excel (Microsoft Corporation, Redmond, WA) data including: hospital geographical location, foodservice model (room service; traditional pre‐ordering foodservice system); meal preparation model (cook fresh only; predominantly cook fresh); number of meals served per day; menu type (full ward diet for children, full ward diet for adults); meal period (breakfast, morning tea, lunch, afternoon tea, dinner, supper); time of the meal service; foods and dishes description, identification of options (e.g., patients could choose from different types of hot meals during lunch), number of serves or amount (in grams or millilitres); recipe of dishes prepared onsite; product brand and name for packaged products; and identification of UPFs through the presence of markers (i.e., food substance of mostly exclusive industrial use, such as hydrogenated oils, protein isolates, high‐fructose corn syrup, or cosmetic additives, such as flavours, colours, thickeners, emulsifiers) (Data S1).

For UPF identification, dietitians were instructed to record at least one marker of UPF identified in the packaged product, whether the item was a standalone menu item (e.g., fruit drink) or an ingredient within a mixed dish recipe (e.g., mayonnaise in a sandwich). These were cross‐checked for accuracy by a trained nutritionist with experience in identifying UPFs. All dietitians completed data collection for standard inpatient menu (full ward diet) offered over seven consecutive days. All data were shared with the research team via a secure drive. An illustrative menu example was provided to participants in the data collection manual (Data S1).

There were several steps to the analysis. First, all foods and drinks recorded per day were imported into FoodWorks P10 Premium software (Xyris Pty Ltd., Brisbane, 2019) to derive the energy content of each menu item. The Food composition databases selected for the nutritional analysis were those available in the FoodWorks P10 Premium software (Xyris Pty Ltd., Brisbane, 2019) and were Australian Food Composition Database 2019, AusBrands 2019, and AusFoods. Individual food items or mixed dishes were categorised into 14 food categories: 1. Bread, 2. Cereal, 3. Dairy, 4. Dessert, 5. Drink, 6. Fruit, 7. Hot option, 8. Spread/Sauce, 9. Cake/Pastry, 10. Snack, 11. Salad, 12. Sandwich, 13. Side, 14. Soup. These categories were used to determine key types of UPFs supplied in hospitals. Table 1 presents the list of food categories and examples.

**TABLE 1:** Categories for classifying food items on hospital menus and examples.^a^

Food category	Examples of foods included in each category
1. Bread	Wholemeal and white sliced bread, loaf and rolls
2. Cereal	Breakfast cereals, muesli, rolled oats
3. Dairy	Milk, cheese, yoghurt (includes drinking yoghurt)
4. Dessert	Pannacotta, ice cream, pudding
5. Drink	Juice, fruit drink, cordial, tea, coffee, high protein drinks (e.g., chocolate milkshake)
6. Fruit	Apple, banana, fruits in syrup
7. Hot option	Scrambled eggs, honey soy chicken, pasta Napoli
8. Spread/Sauce	Marmalade, peanut butter, mayonnaise
9. Cake/Pastry	Chocolate cake, gherkin and cheese scones, sausage rolls
10. Snack	Crackers, nut mix, jelly
11. Salad	Roast turkey salad plate, tuna bean salad, vegetarian garden salad
12. Sandwich	Tuna salad sandwich, ham and cheese sandwich, roast turkey and tomato sandwich
13. Side	Rice, mashed potatoes, green beans
14. Soup	Tomato soup, cream of pumpkin soup, chicken instant noodle soup

^a^ Table includes UPF and non‐UPF examples.

Once all energy content derived from the Foodworks database was calculated by food category within each meal period, these were averaged across 7 days. The median number and proportion of UPF items over 7 days were estimated by food category (e.g., cereal, within hot options) within each meal period (e.g., breakfast), both overall and separately for each hospital. This information can be used to recalculate the total number of items available overall (i.e., UPF and non‐UPF) in that food category and meal period, which was not presented to avoid redundancy. Similarly, the median and interquartile range (IQR, 25th and 75th percentile) of the total energy content (kJ) of each food category (stratified by meal period) was estimated (i.e., including energy from UPF and non‐UPF items), as well as the respective proportion of energy from UPFs. This energy approach was also included to illustrate the proportion of UPFs and their contribution to energy availability per meal, since many UPFs occur multiple times across a menu (e.g., the same bread types can be offered at breakfast, afternoon tea and dinner). The “drink” category was not estimated by meal period, as some hospitals only provided the overall list of drinks available to patients on request throughout the day. The number of oral nutrition support products provided by each hospital, as well as the respective proportion of UPFs, were estimated. The number of hospitals located in metropolitan and regional areas across Australian states, as well as hospital size categories (small: <100 beds; medium: 100–499 beds; large: ≥500 beds), were described. To minimise the risk of re‐identification, only metropolitan/regional location was reported for individual hospitals, while other characteristics were presented in aggregate.

Stata software (Stata Corp., version 18, SE) was used for data analysis. This study is reported using the STROBE (Strengthening the Reporting of Observational Studies in Epidemiology) checklist for cross‐sectional studies (Data S3).

## 3 RESULTS

Out of 20 eligible hospitals invited, 6 agreed to participate, 4 declined, while 10 did not respond. Included hospitals were located in metropolitan (*n* = 5) and regional (*n* = 1) areas across the Australian states of Queensland, Victoria, and South Australia, and were of small (*n* = 1, i.e., <100 beds), medium (*n* = 4, i.e., 100–499 beds) and large (*n* = 1, i.e., ≥500 beds) sizes. All included hospitals had a traditional ordering system, where meals were ordered in advance of the meal period. Most (5 hospitals) had predominantly cook fresh foodservice system (i.e., minimal menu items were not cooked or prepared on site), and the number of meals served in the hospitals per day ranged from 100 to 1200. Hospitals have been de‐identified for this analysis and re‐coded as Hospitals 1–6 (in no particular order).

Table 2 presents the median number and proportion of UPF items, total energy content (i.e., considering all UPF and non‐UPF items) and proportion of energy from UPFs by food categories within meal period, on full ward hospital menus. Bread available for breakfast and sandwiches available for dinner are here used as examples to support with table interpretation. Across all hospitals, the median number of ultra‐processed bread options available for breakfast was 3, representing 90% of all bread options offered at breakfast (see Table 2, column “UPF items, *N*(%)”). The median energy content of all bread options available for breakfast was 467 kJ (IQR: 371–542 kJ) (Table 2, column “Energy (kJ), Median (IQR)”). Of these, UPF breads accounted for 88% of total energy content (Table 2, column “% Energy from UPFs, Median (IQR)”). Similarly, hospitals provided disaggregated information of the ingredients of sandwiches offered for dinner. The contribution of UPF and non‐UPF items within those sandwiches was estimated (e.g., tuna sandwich was disaggregated and classified according to its ingredients: bread, canned tuna, mayonnaise, celery, pepper, pickles, and margarine). Across all hospitals, the median number of ultra‐processed foods that were ingredients in sandwiches available for dinner was 7, representing 57% of all ingredients in sandwiches offered at dinner. The median energy content of all entire sandwich options available for dinner was 1502 kJ (IQR: 1327–1668 kJ). Of these, UPF ingredients accounted for 69% of total energy content (Table 2). This approach to interpreting ingredients within recipes was also applied to other categories comprising mixed dishes prepared on site (e.g., desserts, hot options, cakes/pastries, salads, sandwiches, sides, and soups).

**TABLE 2:** Median number and proportion of ultra‐processed food options (or ingredients in mixed dishes), total energy content (combined ultra‐processed and non‐ultra‐processed food items), and contribution of ultra‐processed foods to total energy content, by food category within meal periods on full ward hospital menus (*n* = 6 hospitals over 7 days).

Meal period/food category	UPF items^a^	Energy (combined UPF and non‐UPF) (kJ)^b^	% Energy from UPFs^c^
	*N* (%)	Median (IQR)	Median (IQR)
**Breakfast**			
Bread	3 (90)	467 (371–542)	88 (88–88)
Cereal	3 (64)	516 (485–595)	67 (67–67)
Dairy	1 (11)	432 (277–647)	10 (10–19)
Dessert	3 (100)	605 (605–605)	100 (100–100)
Fruit	0 (0)	302 (242–343)	0 (0–0)
Hot option	2 (37)	1016 (952–1267)	39 (36–52)
Spread/Sauce	7 (69)	157 (122–281)	64 (63–64)
**Morning tea**			
Bread	3 (100)	358 (292–434)	100 (100–100)
Cake/Pastry	2 (35)	674 (542–977)	45 (13–85)
Dairy	1 (26)	237 (222–290)	29 (28–29)
Fruit	1 (11)	400 (318–458)	15 (15–15)
Snack	3 (78)	314 (267–420)	83 (80–83)
Spread/Sauce	4 (52)	164 (143–218)	41 (41–41)
**Lunch**			
Bread	2 (92)	331 (304–352)	91 (91–91)
Dairy	0.5 (26)	305 (258–362)	26 (26–26)
Dessert	5 (80)	406 (293–547)	88 (68–89)
Fruit	0 (0)	272 (195–360)	0 (0–0)
Hot option	3 (30)	1379 (1023–1805)	24 (10–35)
Salad	2 (21)	591 (530–856)	31 (13–40)
Sandwich	6 (57)	1492 (1385–1702)	78 (55–85)
Spread/Sauce	6 (80)	233 (193–274)	79 (79–79)
Side	8 (19)	306 (174–324)	9 (1–32)
Soup	2 (42)	298 (323–310)	42 (30–70)
**Afternoon tea**			
Bread	3 (100)	358 (292–434)	100 (100–100)
Dairy	1 (25)	275 (244–343)	27 (27–27)
Fruit	1 (10)	380 (310–436)	11 (11–11)
Sandwich	37 (47)	1534 (1360–1855)	85 (77–95)
Snack	3 (77)	320 (252–454)	80 (79–80)
Spread/Sauce	5 (75)	221 (95–269)	70 (70–70)
**Dinner**			
Bread	2 (75)	404 (377–420)	75 (75–75)
Dairy	0.3 (11)	277 (205–365)	11 (11–11)
Dessert	5 (74)	438 (314–586)	79 (59–88)
Fruit	0.2 (3)	304 (221–382)	0 (0–9)
Hot option	3 (22)	1378 (932–1685)	20 (6–45)
Salad	1 (15)	278 (269–517)	25 (19–40)
Sandwich	7 (57)	1502 (1327–1668)	69 (53–75)
Side	2 (14)	254 (116–399)	6 (0.3–21)
Spread/Sauce	5.0 (74)	206 (181–234)	72 (72–73)
Soup	8 (53)	1038 (719–1790)	51 (42–58)
**Supper**			
Bread	3 (100)	358 (292–434)	100 (100–100)
Dairy	1 (22)	319 (212–421)	26 (26–26)
Fruit	0.5 (8)	370 (277–415)	11 (11–11)
Sandwich	37 (47)	1534 (1360–1855)	85 (77–95)
Snack	3 (83)	393 (265–449)	85 (84–85)
Spread/Sauce	4 (50)	164 (143–218)	41 (41–41)

*Note*: Full ward hospital menus: does not include drinks, these are presented in Table 4.

Abbreviation: IQR, interquartile range, 25th and 75th percentile.

^a^ Presents the median number of UPF options within the specific food category and meal period; % refers to the number of UPF items as a proportion of the total number of food items. It also reflects the number of UPF ingredients in mixed dishes (e.g., sandwiches) and their proportion of the total number of food items.

^b^ Calculated as the median energy content of all items belonging to the food category, i.e., both UPF and non‐UPF items.

^c^ Calculated as the contribution of UPFs (%) to the energy content (kJ) of all items belonging to the food category.

**FIGURE 1:**
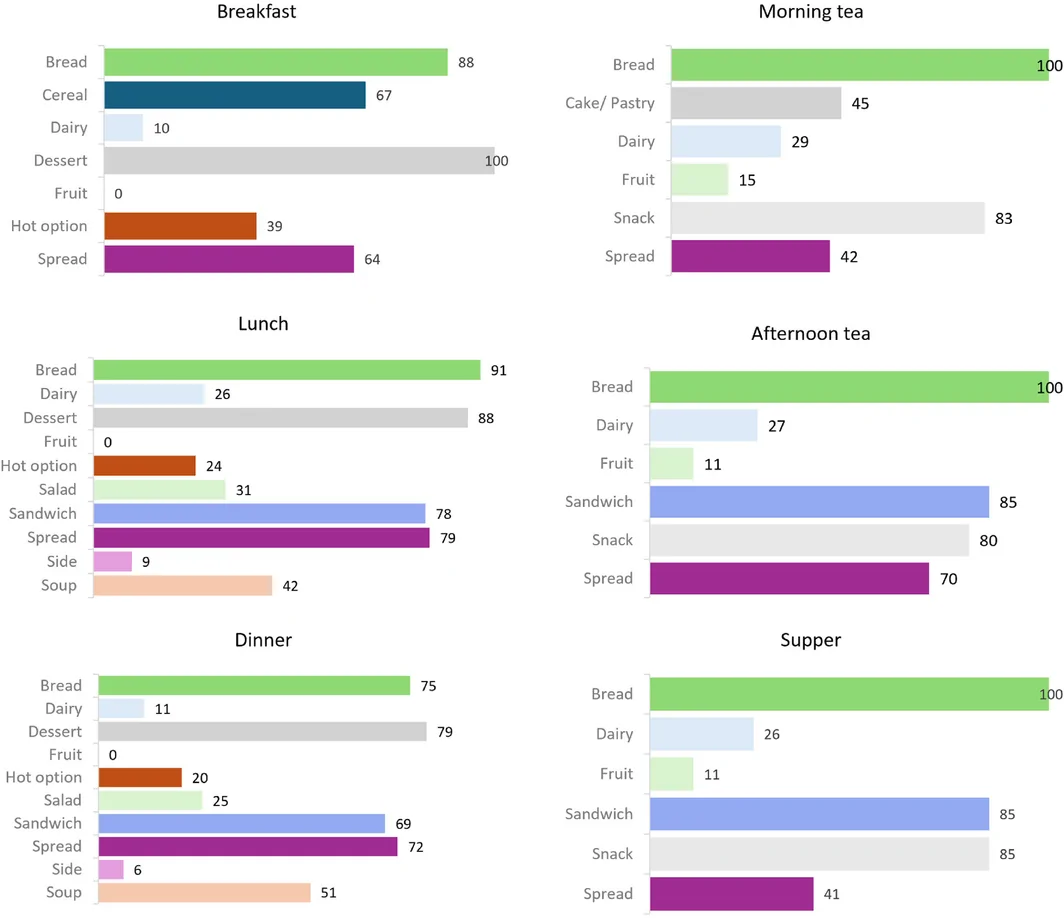
Median proportion of energy from ultra‐processed foods across meal periods and food categories (*n* = 6 hospitals).

Figure 1 presents the median proportion of energy from UPFs in a visual manner. UPFs contributed to a significant proportion of the items and energy across all meal periods, with 4 out of 7 food categories at breakfast having >50% of energy from UPFs; 2 out of 6 food categories at morning tea, 4 out of 10 at lunch, 4 out of 6 at afternoon tea, 5 out of 10 at dinner, and 3 out of 6 at supper (Table 2 and Figure 1).

Bread, dessert (e.g., ready‐to‐eat vanilla custard and pudding, ice cream), snack (e.g., biscuits, crackers, jelly), and spread/sauce (e.g., margarine, mayonnaise, barbeque sauce) were the food categories with the highest proportion of UPF items across all meal periods, with over two‐thirds of offered items classified as UPFs. However, a higher proportion of energy contribution from UPFs were observed for cereals at breakfast (67%) and sandwiches at afternoon tea and supper (85% for both meals). This shows that despite a smaller proportion of UPF items, they contribute to a significantly higher proportion of energy within these food categories (e.g., tuna sandwich has most of its energy content from UPF bread and mayonnaise). Fruits, side dishes, dairy and hot options were the food categories with the lowest energy contribution from UPFs (Table 2 and Figure 1).

Table 3 presents similar data stratified by hospital. Findings show the proportion of energy contribution from UPFs varies across hospitals, particularly for cereals—varying from 0% in hospital 6 to 100% in hospitals 2 and 3, and hot options—varying from 0–1% in hospitals 2 and 5 to 100% in hospital 3. It also varies within hospitals, for example sandwiches from 59% to 98% in dinner vs. lunch, respectively, at hospital 5. Hot options and sandwiches were the food categories with the higher amount of energy content.

**TABLE 3:** Median number and proportion of ultra‐processed food items (or ingredients in mixed dishes), total energy content (combined ultra‐processed and non‐ultra‐processed food items), and contribution of ultra‐processed foods to total energy content, by food categories within meal periods and hospital (*n* = 6 hospitals over 7 days).

Meal period/food category	Hospital 1 (regional)			Hospital 2 (metropolitan)			Hospital 3 (metropolitan)		
	UPF items^a^	Energy (combined UPF and non‐UPF) (kJ)^b^	% Energy from UPFs^c^	UPF items^a^	Energy (combined UPF and non‐UPF) (kJ)^b^	% Energy from UPFs^c^	UPF items^a^	Energy (combined UPF and non‐UPF) (kJ)^b^	% Energy from UPFs^c^
	*N* (%)	Median (IQR)	Median (IQR)	*N* (%)	Median (IQR)	Median (IQR)	*N* (%)	Median (IQR)	Median (IQR)
**Breakfast**									
Bread	3 (100)	798 (644–864)	100 (NV)	3 (100)	358 (292–434)	100 (NV)	3 (75)	346 (311–362)	73 (NV)
Cereal	4 (67)	740 (678–795)	74 (NV)	4 (100)	498 (476–605)	100 (NV)	6 (100)	472 (467–570)	100 (NV)
Dairy	0 (0)	434 (328–672)	0 (0)	1 (23)	537 (353–679)	32 (NV)	1 (22)	352 (311–682)	15 (0–55)
Dessert	‐	‐	‐	‐	‐	‐	3 (100)	86 (86–433)	100 (NV)
Fruit	‐	‐	‐	0 (0)	386 (282–407)	0 (NV)	0 (0)	267 (182–330)	0 (NV)
Hot option	2 (50)	1626 (NV)	50 (NV)	2 (24)	370 (289–1015)	42 (NV)	2 (87)	544 (382–1119)	100 (88–100)
Spread	7 (87)	56 (54–123)	90 (87–90)	4 (50)	164 (144–218)	41 (NV)	13 (81)	148 (70–259)	82 (NV)
**Morning tea**									
Bread	‐	‐	‐	3 (100)	358 (292–434)	100 (NV)	‐	‐	‐
Cake/Pastry	2 (30)	1054 (903–1261)	33 (13–77)	2 (40)	295 (180–692)	58 (14–93)	‐	‐	‐
Dairy	‐	‐	‐	2 (33)	400 (326–663)	46 (40–47)	1 (100)	328 (NV)	100 (NV)
Fruit	‐	‐	‐	2 (33)	396 (282–506)	46 (NV)	‐	‐	‐
Snack	‐	‐	‐	4 (76)	371 (361–426)	80 (67–80)	7 (64)	384 (355–665)	66 (NV)
Spread	‐	‐	‐	4 (52)	164 (144–218)	41 (NV)	‐	‐	‐
**Lunch**									
Bread	‐	‐	‐	2 (100)	325 (292–358)	100 (NV)	3 (75)	346 (311–362)	73 (NV)
Dairy	1 (100)	348 (NV)	100 (NV)	0 (0)	321 (295–400)	0 (NV)	1 (57)	297 (274–328)	55 (NV)
Dessert	2 (26)	208 (174–226)	41 (13–43)	5 (100)	599 (527–660)	100 (NV)	9 (100)	537 (411–574)	100 (NV)
Fruit	0 (0)	280 (84–407)	0 (0)	0 (0)	282 (268–386)	0 (NV)	0 (0)	267 (190–330)	0 (NV)
Hot option	3 (25)	1412 (1070–1483)	8 (1–37)	5 (24)	698 (352–1223)	1 (0–21)	1 (75)	1235 (899–1758)	100 (50–100)
Salad	‐	‐	‐	0.5 (2)	873 (690–1275)	0 (NV)	3 (41)	1018 (1017–1613)	62 (17–66)
Sandwich	‐	‐	‐	10 (57)	1278 (1155–1393)	67 (48–76)	6 (66)	1520 (1386–1955)	78 (56–82)
Spread	1 (100)	491 (353–629)	100 (NV)	‐	‐	‐	12 (80)	148 (70–259)	82 (NV)
Side	2 (23)	573 (385–770)	23 (3–45)	3 (19)	445 (243–601)	0 (0–28)	2 (42)	258 (104–323)	33 (0–100)
Soup	‐	‐	‐	‐	‐	‐	1 (88)	299 (347–429)	100 (76–100)
**Afternoon tea**									
Bread	‐	‐	‐	3 (100)	358 (292–434)	100 (NV)	‐	‐	‐
Dairy	1 (23)	466 (357–606)	20 (NV)	2 (29)	400 (321–663)	40 (NV)	1 (100)	328 (NV)	100 (NV)
Fruit	0 (0)	320 (284–369)	0 (0)	2 (33)	396 (282–506)	46 (NV)	‐	‐	‐
Sandwich	37 (47)	1534 (1360–1855)	87 (77–95)	‐	‐	‐	‐	‐	‐
Snack	4 (78)	528 (230–621)	65 (NV)	4 (78)	382 (361–426)	80 (NV)	7 (64)	384 (355–665)	66 (NV)
Spread	‐	‐	‐	4 (50)	164 (144–218)	41 (NV)	5 (100)	277 (47–320)	100 (NV)
**Dinner**									
Bread	‐	‐	‐	2 (100)	325 (251–358)	100 (NV)	3 (75)	346 (311–362)	73 (NV)
Dairy	‐	‐	‐	0.2 (3)	321 (321–400)	0 (NV)	1 (53)	297 (265–328)	55 (NV)
Dessert	2 (33)	87 (NV)	26 (NV)	5 (100)	580 (526–608)	100 (NV)	10 (99)	507 (486–527)	100 (NV)
Fruit	‐	‐	‐	0 (0)	282 (265–386)	0 (NV)	1 (11)	267 (182–330)	0 (0–38)
Hot option	3 (28)	2197 (1640–3138)	48 (8–87)	6 (20)	852 (523–1221)	4 (0–34)	3 (48)	967 (381–1020)	44 (25–100)
Salad	1 (14)	289 (NV)	64 (NV)	1 (4)	690 (690–1268)	0 (0–3)	0.3 (9)	8 (8–597)	0 (0–54)
Sandwich	‐	‐	‐	9 (65)	1339 (1203–1403)	67 (57–77)	‐	‐	67 (57–77)
Side	2 (17)	574 (121–620)	23 (1–76)	3 (17)	461 (176–601)	0 (0–30)	2 (22)	88 (18–323)	0 (0–0)
Spread	‐	‐	‐	‐	‐	‐	15 (85)	146 (70–241)	85 (83–86)
Soup	3 (28)	565 (535–981)	40 (0–51)	‐	‐	‐	1 (100)	693 (172–2209)	100 (NV)
**Supper**									
Bread	‐	‐	‐	3 (100)	358 (292–434)	100 (NV)	‐	‐	‐
Dairy	1 (25)	466 (360–606)	20 (NV)	2 (29)	400 (325–663)	40 (NV)	1 (33)	328 (82–338)	44 (NV)
Fruit	0 (0)	320 (284–369)	0 (0)	2 (33)	396 (282–506)	46 (NV)	0 (0)	359 (136–377)	0 (NV)
Sandwich	37 (47)	1534 (1360–1855)	87 (77–95)	‐	‐	‐	‐	‐	‐
Snack	4 (80)	528 (230–621)	65 (NV)	4 (78)	382 (361–426)	80 (NV)	3 (76)	373 (254–384)	95 (94–95)
Spread	‐	‐	‐	4 (50)	164 (144–218)	41 (NV)	‐	‐	‐

Meal period/food category	Hospital 4 (metropolitan)			Hospital 5 (metropolitan)			Hospital 6 (metropolitan)		
	UPF items^a^	Energy (combined UPF and non‐UPF) (kJ)^b^	% Energy from UPFs^c^	UPF items^a^	Energy (combined UPF and non‐UPF) (kJ)^b^	% Energy from UPFs^c^	UPF items^a^	Energy (combined UPF and non‐UPF) (kJ)^b^	% Energy from UPFs^c^
	*N* (%)	Median (IQR)	Median (IQR)	*N* (%)	Median (IQR)	Median (IQR)	*N* (%)	Median (IQR)	Median (IQR)
**Breakfast**									
Bread	3 (75)	533 (323–743)	66 (NV)	‐	‐	‐	2 (100)	297 (283–311)	100 (NV)
Cereal	2 (40)	477 (474–608)	41 (NV)	4 (80)	477 (474–520)	87 (NV)	0 (0)	430 (342–473)	0 (NV)
Dairy	1 (25)	526 (240–854)	30 (NV)	0 (0)	489 (321–656)	0 (NV)	0 (0)	234 (111–339)	0 (NV)
Dessert	‐	‐	‐	‐	‐	‐	‐	‐	‐
Fruit	0 (0)	282 (259–310)	0 (NV)	0 (0)	323 (259–386)	0 (NV)	0 (0)	254 (230–284)	22 (NV)
Hot option	1 (12)	2021 (2021–2056)	7 (NV)	0.5 (13)	519 (439–519)	0 (0–60)	‐	‐	‐
Spread	4 (67)	169 (147–210)	59 (NV)	5 (62)	249 (164–642)	60 (NV)	6 (67)	159 (152–236)	55 (NV)
**Morning tea**									
Bread	‐	‐	‐	‐	‐	‐	‐	‐	‐
Cake/Pastry	‐	‐	‐	‐	‐	‐	‐	‐	‐
Dairy	0 (0)	80 (NV)	0 (NV)	0 (0)	340 (NV)	0 (NV)	0 (0)	38 (NV)	0 (NV)
Fruit	0 (0)	407 (NV)	0 (NV)	0 (0)	396 (266–461)	0 (NV)	‐	‐	‐
Snack	2 (100)	290 (216–364)	100 (NV)	2 (100)	234 (216–253)	100 (NV)	1 (50)	290 (189–392)	67 (NV)
Spread	‐	‐	‐	‐	‐	‐	‐	‐	‐
**Lunch**									
Bread	2 (100)	323 (309–337)	100 (NV)	‐	‐	‐			
Dairy	0 (0)	400 (NV)	0 (NV)	0 (0)	424 (193–656)	0 (NV)	0 (0)	38 (NV)	0 (NV)
Dessert	2 (79)	659 (472–807)	100 (43–100)	3 (100)	404 (253–421)	100 (NV)	6 (72)	386 (337–543)	88 (50–97)
Fruit	0 (0)	347 (308–386)	0 (NV)	0 (0)	182 (123–290)	0 (NV)	‐	‐	‐
Hot option	3 (16)	2134 (1632–2841)	11 (1–17)	3 (8)	1249 (1052–1396)	0 (0–2)	4 (25)	1490 (967–1739)	26 (5–35)
Salad	2 (29)	308 (275–327)	44 (31–60)	‐	‐	‐	2 (14)	168 (140–210)	19 (6–36)
Sandwich	7 (70)	2136 (1859–2355)	84 (65–89)	5 (71)	1447 (1447–1585)	98 (82–100)	2 (25)	1078 (1078–1224)	62 (24–81)
Spread	2 (60)	198 (157–210)	55 (NV)	‐	‐	‐	‐	‐	‐
Side	1 (14)	212 (63–813)	0 (NV)	0.5 (12)	111 (98–152)	0 (0–21)	1 (7)	235 (149–238)	0 (0–0)
Soup	2 (23)	2060 (1591–2312)	19 (15–28)	2 (29)	750 (515–1012)	7 (0–82)	‐	‐	‐
**Afternoon tea**									
Bread	‐	‐	‐	‐	‐	‐	‐	‐	‐
Dairy	0 (0)	0 (NV)	0 (NV)	0 (0)	340 (NV)	0 (NV)	0 (0)	38 (NV)	0 (NV)
Fruit	0 (0)	0 (NV)	0 (NV)	0 (0)	396 (266–461)	0 (NV)	‐	‐	‐
Sandwich	‐	‐	‐	‐	‐	‐	‐	‐	‐
Snack	2 (100)	2 (100–290)	100 (NV)	2 (100)	234 (216–253)	100 (NV)	1 (43)	101 (138–392)	67 (65–67)
Spread	‐	‐	‐	‐	‐	‐	‐	‐	‐
**Dinner**									
Bread	2 (100)	323 (309–337)	100 (NV)	0 (0)	730 (NV)	0 (NV)	2 (100)	297 (283–311)	100 (NV)
Dairy	0 (0)	240 (80–400)	0 (NV)	0 (0)	489 (321–656)	0 (NV)	0 (0)	38 (NV)	0 (NV)
Dessert	3 (61)	711 (585–1064)	92 (30–100)	4 (100)	411 (408–549)	100 (NV)	6 (48)	636 (286–1005)	57 (0–100)
Fruit	0 (0)	357 (307–407)	0 (NV)	0 (0)	308 (128–407)	0 (NV)	‐	‐	‐
Hot option	2 (12)	2249 (1623–3012)	5 (0–29)	1 (3)	1370 (1212–1436)	0 (0–0)	4 (19)	1191 (944–1590)	19 (2–21)
Salad	2 (29)	308 (275–327)	44 (31–60)	‐	‐	‐	1 (19)	93 (81–106)	18 (0–21)
Sandwich	7 (70)	2136 (1859–2355)	84 (65–89)	9 (69)	1458 (1187–1754)	59 (49–66)	4 (33)	1078 (1059–1159)	66 (40–69)
Side	1 (14)	68 (62–487)	0 (NV)	1 (11)	101 (84–122)	16 (1–19)	0.5 (3)	235 (235–244)	0 (0–0)
Spread	3 (60)	198 (177–210)	55 (NV)	1 (100)	243	100 (NV)	1 (50)	239 (236–241)	50 (NV)
Soup	2 (16)	1933 (1251–2620)	15 (8–37)	1 (100)	1254 (NV)	100 (NV)	2 (19)	747 (385–1887)	2 (0–3)
**Supper**									
Bread	‐	‐	‐	‐	‐	‐	‐	‐	‐
Dairy	0 (0)	80	0 (NV)	‐	‐	‐	‐	‐	‐
Fruit	0 (0)	407	0 (NV)	‐	‐	‐	‐	‐	‐
Sandwich	‐	‐	‐	‐	‐	‐	‐	‐	‐
Snack	2 (100)	290 (216–364)	100 (NV)	‐	‐	‐	‐	‐	‐
Spread	‐	‐	‐	‐	‐	‐	‐	‐	‐

*Note*: Does not include drinks; these are presented in Table 4.

Abbreviations: IQR, interquartile range, 25th and 75th percentile; NV, no variation in options across days.

^a^ Presents the median number of UPF options within the specific food category and meal period; % refers to the number of UPF items as a proportion of the total number of food items. It also reflects the number of UPF ingredients in mixed dishes (e.g., sandwiches) and their proportion of the total number of food items.

^b^ Calculated as the median energy content of all items belonging to the food category, i.e., both UPF and non‐UPF items.

^c^ Calculated as the contribution of UPFs (%) to the energy content (kJ) of all items belonging to the food category.

Table 4 presents the number and proportion of UPF items, energy content, and proportion of energy from UPFs of the category of drinks, overall and by hospital. Sixty‐one percent of drink items were classified as UPFs, contributing to 76% of energy from UPFs within this category. A large variation was observed across hospitals, varying from 9% in hospital 6 to 87% and 88% in hospitals 2 and 1, respectively.

**TABLE 4:** Median number and proportion of ultra‐processed food items, total energy content (combined ultra‐processed and non‐ultra‐processed food items), and contribution of ultra‐processed foods to total energy content of the category of drinks, on full ward hospital menus (*n* = 6 hospitals over 7 days).

	UPF items^a^	Energy (combined UPF and non‐UPF) (kJ)^b^	% Energy from UPFs^c^
	*N* (%)	Median (IQR)	Median (IQR)
All	5 (61)	157 (93–229)	76 (54–88)
Hospital 1	16 (84)	247 (3–308)	88 (NV)
Hospital 2	9 (59)	287 (115–491)	87 (75–91)
Hospital 3	18 (64)	139 (87–318)	65 (NV)
Hospital 4	2 (40)	77 (66–185)	50 (NV)
Hospital 5	2 (100)	174 (140–208)	100 (NV)
Hospital 6	0.2 (7)	23 (17–37)	9 (NV)

*Note*: Does not include milk and drinking yoghurt (accounted within “dairy” in Tables 2 and 3).

Abbreviations: IQR, interquartile range, 25th and 75th percentile; NV, no variation in options across days.

^a^ Presents the median number of UPF options within the drink category; % refers to the number of UPF items as a proportion of the total number of drink items.

^b^ Calculated as the median energy content of all items belonging to the drink category, i.e., both UPF and non‐UPF items.

^c^ Calculated as the contribution of UPFs (%) to the energy content (kJ) of all items belonging to the drink category.

Considering the contribution of both foods and drinks across all hospitals, UPFs contributed to 47% of the items and 51% of total energy (data not shown). Oral nutrition support products reported by hospitals ranged from 7 to 13 options; all were categorised as UPF (data not shown) due to their ingredients that include thickeners, flavours, maltodextrin, protein isolates, among other UPF markers. Due to inconsistencies in reporting short‐order menu across hospitals, these were not included in the analyses.

## 4 DISCUSSION

This study analysed the supply of UPFs in a sample of six Australian hospitals using cook fresh foodservice systems. UPFs contributed significantly to total energy available from all menu items (51%) and across all meal periods, with breakfast and afternoon tea having the largest proportion of enegy from UPFs. Bread, dessert, snack, spread/sauce, and drinks were the food categories with the largest number of items classified as UPFs. Hot options and sandwiches were the food categories with the higher amount of energy content but differed substantially in the proportion derived from UPFs. UPFs contributed around 25% of energy in hot options, compared with more than 80% in sandwiches.

To the best of our knowledge, this is the first study to estimate the supply of UPFs in a sample of Australian hospitals, and the first study internationally to include a sample size >2 hospitals, adding to the limited evidence on UPF supply in healthcare settings. Studies to date conducted in Brazil and Greece have found a much lower contribution of UPFs in comparison to our study.^30^, ^31^, ^32^ In Brazil, UPFs contributed 10% and 14% of the total number of items in the menus of two hospitals attending adults and children, respectively (contribution to total energy was not measured).^30^, ^31^ The study involving one hospital in Greece estimated that UPFs contributed 25% of total energy.^32^ Similar to ours, previous analyses did not include the contribution of oral nutritional supplements in the main analysis of the standard inpatient menu since these are not part of the standard hospital diet and are often used to improve or maintain nutritional status of certain populations.^33^ Considering this, we have presented the analysis of oral nutritional supplements separately. These items are mostly UPFs (as found in our analysis), and therefore the median UPF contribution to energy intake will have been underestimated in the menu of patients where they have been prescribed.

Drivers of UPF supply in Australian hospitals have been reported previously.^34^ Hospital menu and nutrition standards, food procurement and contracts lacking food processing criteria (or favouring bulk procurement contracts for UPFs), cost, convenience, shelf life, and workforce capacity have been suggested as reasons for reliance on UPFs,^34^ which overlap with drivers of UPF consumption.^30^, ^35^ Hospitals have unique food environments, where meals must meet clinical and dietary requirements, while accommodating patient preferences. Particularly in this context, UPFs can be favoured because they are cost‐effective, have extended shelf lives, and reduce the need for skilled labour and preparation time. Standardised, pre‐packaged UPF items also support consistency and simplify logistics across large hospital systems, whereas certain UPFs may be used to meet diverse dietary requirements (e.g., portion‐controlled sodium meals).^28^, ^29^, ^30^, ^34^ They are also perceived as convenient, particularly in settings with constrained staffing, equipment, storage, and preparation space, where large volumes of food must be produced and delivered quickly.^34^ Healthcare foodservice is also known to be a challenging environment to implement strategies that are environmentally sustainable,^26^, ^28^ therefore making the reduction of their UPF supply a complex task. While motivated individuals and dedicated personnel, supported by leadership and protocols, can support implementation of healthy and sustainably favourable changes, inadequate policy direction and staff resistance have been reported as deterrents to such initiatives.^28^

While evidence on the role of overall and specific UPFs in the diet continues to emerge,^36^ the adverse human,^8^ and planetary health^16^ impacts associated with increased UPFs supply and consumption should not be neglected. These impacts have important implications for food environments, including hospitals.^37^ Healthcare settings have a fundamental responsibility to provide care and model healthy practices including nutrition to people who are frequently living with chronic disease. In Australia, the importance of a “climate in all health policies” approach—including within the health system—has been nationally recognised as vital to addressing the impacts of climate change and other environmental challenges.^38^ In 2022–2023, 4.5 million patients received overnight hospital care in Australia,^39^ underscoring the significant reach and influence of hospital settings.

The role of policies and interventions in improving institutional settings has been increasingly recognised.^23^, ^24^ The World Health Organization released key frameworks to guide governments in implementing policies, such as food procurements, to promote healthy and sustainable foods in institutional settings, including hospitals.^24^, ^40^ Yet, there is a gap between international and Australian policy recommendations on healthy and environmentally sustainable foodservices and their translation into practice in this sector.^29^ Currently, no federal or state‐level food service or procurement policies in Australia provide recommendations on UPFs in public institutions. For example, while the “Nutrition and quality food standards for adults in Victorian public hospitals and residential aged care services”^41^ include restrictions on items such as processed meats and salty snacks, they do not offer guidance on limiting UPFs, including total amount or proportion of the diet/menu. These nutrition standards play a crucial role in guiding the types and variety of foods offered, establishing minimum choice requirements and nutrient targets, and promoting patient choice while accommodating cultural and clinical needs. However, they could be strengthened to account for the impacts of food ultra‐processing on the overall quality of foods provided, while still ensuring menus meet minimum nutrient requirements for nutritional adequacy.

Findings from this study can inform future policy actions, such as food procurement and nutrition standards, and support targeted interventions to improve the quality of foods provided in Australian hospitals.^37^ This was identified by Australian experts as a research priority to address the harms of UPFs in the country.^42^ They can also guide immediate revisions to budget allocations and meal planning practices. Despite large variation across hospitals, our findings and of others^30^, ^31^ highlight common opportunities to improve the food provision and reduce UPF supply in hospitals. Breakfast and afternoon tea are the main meals providing UPFs, particularly through the supply of UPF breads, snacks, spreads, and drinks, suggesting that modifying these items could lead to substantial improvements in menu quality. UPF supply at lunch and dinner—meals that provide the highest energy content—is relatively low overall; however, patients often choose between a low‐UPF hot meal and a high‐UPF sandwich. Ideally, reducing UPFs in sandwiches (e.g., replace with non‐UPF types of bread, sauce, and meat) could further lower UPF supply. Future research could help identify the specific drivers of UPFs in hospitals outside Australia, trade‐offs and strategies to reduce UPF supply while promoting unprocessed and minimally processed foods.

This is the first study to present analysis of the UPF supply in a sample of Australian hospitals. Trained dietitians collected comprehensive information to identify UPFs (Data S1) over seven consecutive days, including disaggregated ingredients in recipes of mixed dishes. Energy estimation was based on the most up to date Australian food composition databases. The inclusion of hospitals with cook fresh systems provided an opportunity to more accurately assess UPFs, as ingredients and recipe components are prepared on‐site and available locally. Nevertheless, the findings of this study should be generalised to all Australian hospitals with caution. The sample included only hospitals using predominantly (or exclusively) cook fresh, likely providing a lower proportion of UPF items than hospitals operating cook‐chill foodservice systems. While the sample size is limited, it is larger than in similar studies,^30^, ^31^, ^32^ which often include only 1‐2 hospitals. Small samples are common in research on hospital food supply and menu composition, as data collection is resource‐intensive and requires direct access to procurement and menu information. Future research could explore ways to streamline data collection and build on this exploratory evidence as a foundation for larger‐scale studies.

Although a large variation in the production methods is observed in Australia,^43^ some regions have become reliant on cook‐chill foodservice systems.^44^ The decision to adopt such systems ensures that the receiving hospital becomes a finishing kitchen, heavily reliant on external suppliers for partial or complete preparation of foods, which impacts UPF supply. Other limitations should be considered. The actual UPF consumption by inpatients was not measured; therefore, it remains uncertain whether, despite the high availability of UPFs, patients predominantly chose non‐UPF alternatives. Short‐order menu items were excluded from the analysis due to inconsistent reporting across hospitals, which may have led to either under or overestimation of UPF supply. Although data were collected over multiple days, the study period may not reflect seasonal variations or supply‐related changes in menu offerings. Finally, potential inaccuracies in completing data collection forms (including in the identification of UPFs) or matching with food composition databases cannot be ruled out.

In conclusion, this study provides novel evidence on the extent of UPF supply in a sample of Australian hospitals that use a cook fresh model. UPFs contributed significantly to total energy overall (51%) and across all meal periods, with breakfast and afternoon tea having the largest proportion of UPFs. These findings can inform Australian policies (e.g., food procurement and nutrition standards) and targeted interventions to improve the quality of foods provided by Australian healthcare foodservices.

## AUTHOR CONTRIBUTIONS

JP and ML conceptualised the study. JP, ML, MF, and RP acquired funding. PM, JP, ML, MF, and RP designed the research. PM had primary responsibility for data collection training and instruments development, data analysis and interpretation. NC supported with data extraction and analysis. PM drafted the manuscript. NC, ML, MF, RP, and JP provided critical revision of the manuscript for important intellectual content; and all authors read and approved the final manuscript. The authors would like to thank the participating hospitals and the dietitians who collected the data.

## FUNDING INFORMATION

This work was supported by a Deakin University IPAN seed fund (Project ID: PJ04610) and a Deakin Sustainable Health Network seed fund (Project ID: PJ04988). PM is supported by a National Health and Medical Research Council (NHMRC) Emerging Leadership Investigator Grant (APP2034008). Supporting source had no involvement or restrictions regarding this research.

## CONFLICT OF INTEREST STATEMENT

Judi Porter is the Editor‐in‐Chief of Nutrition & Dietetics and was excluded from the peer review process and all decision‐making regarding this article. This manuscript has been managed throughout the review process by the Journal's Editor. The Journal operates a blinded peer review process and the peer reviewers for this manuscript were unaware of the authors of the manuscript. This process prevents authors who also hold an editorial role to influence the editorial decisions made. All other authors have no conflicts of interest to declare.

## ETHICS STATEMENT

This study received ethics approval from the Deakin University Human Ethics Advisory Group (HEAG‐H 97_2023). All participants provided informed consent before taking part in the study.

## Supporting information


**Data S1.** Supporting Information.

## Data Availability

Research data are not shared.
