# Supplementary material for: Ultra‐processed food supply in hospitals: A cross‐sectional analysis of Australian hospitals using cook fresh foodservices
Source: Nutr Diet. 2026 Jun 4;83(4):476–89. doi: 10.1111/1747-0080.70083 (PMC13575712; doi:10.1111/1747-0080.70083)
Supplement: Supplementary file 1 [file NDI-83-476-s001.pdf]

Supplementary material

Supplementary material 1. Manual for data collection

Supplementary material 2. Data collection instruments

Supplementary material 3. STROBE checklist for cross-sectional studies

Supplementary material 1.

Contribution of ultra-processed foods in a sample of Australian hospitals

Data collection manual

Research team: Prof. Mark Lawrence (Deakin University), Prof. Judi Porter (Deakin University), Dr Rebecca Patrick (University of Melbourne), Dr Mike Forrester (Deakin University), Dr Priscila Machado (Deakin University)

Contact details: Dr Priscila Machado: (p.machado@deakin.edu.au)

Deakin University ethics approval: Project number HEAG-H 97_2023

22 September 2023

Contents

1. Background and Purpose 3

2. Timeframe 4

2.1 Key dates 4

2.2 Extensions to Timeframe 4

3. Data collection 5

3.1 Inclusion and exclusion criteria 5

3.2 Description of data to be collected 5

3.2.1 Ultra-processed food markers 7

3.2.2 Examples 9

3.3 Data collection procedures 11

4. Additional information 12

1. Background and Purpose

Ultra-processed foods are defined within the NOVA classification system as '*formulations of ingredients, mostly of exclusive industrial use, that result from a series of industrial processes*' and contain little or no whole foods (Monteiro et al., 2019). Ultra-processed food consumption is increasing nearly everywhere, including in Australia (Baker et al., 2020). The consumption of these foods have been associated with a range of adverse health outcomes, including weight gain, type-2 diabetes, cardiovascular diseases, depression, cancer and mortality (Lane et al., 2022).

For this reason, they became an important focus for research and policy, including in studies of food environments, such as the ones in healthcare settings. The proportion of ultra-processed foods available within hospital inpatient menus has been previously assessed in the U.S. (Detopoulou et al., 2022) and Brazil (Zhart et al., 2022; Padovan et al, 2023), but not in Australia.

This study will extend the scope of the existing ultra-processed foods research within Deakin's Institute for Physical Activity and Nutrition into healthcare settings. We conducted a scoping review of the literature (Stephens et al., 2021) and found a gap between international and Australian policy recommendations on healthy and environmentally sustainable foodservices and their translation into practice in this sector.

This project will allow the assessment of the magnitude and drivers of the exposure to ultra-processed foods in hospitals in Australia that will further inform interventions that have potential to strengthen policy recommendations (e.g., food procurement and nutrition standards) and the translation into practice to improve the quality of foods provided by Australian hospitals. This will bring significant health gains for the Australian population.

This project aims to assess and quantify the proportion of ultra-processed foods available within Australian hospital inpatient menus, and determine key types of ultra-processed foods available in each of the menu categories.

2. Timeframe

2.1 Key dates

Key dates for data collection are included in the table below.

Collection activity	Week Commencing
**Signed Consent**	Prior to data collection training
**7 consecutive days data collection**	2nd October – 6th November
**Send Excel spreadsheets to PM**	9th October – 6th November
**Submission completion**	By 6th November 2023
**Destroy hardcopies**	By 30th November 2023

2.2 Extensions to Timeframe

If unable to complete the actions by the prescribed deadlines, they should contact Dr Priscila Machado via p.machado@deakin.edu.au.

3. Data collection

3.1 Inclusion and exclusion criteria

Dietitians (hospitals' representatives) of each included hospital will conduct data collection over 7 consecutive days. The menu is designed on site, and it is standard for those following a "general" diet. The following inclusion and exclusion criteria apply to this project.

**Inclusion**
Standard hospital inpatient menu (Full Ward Diet) for paediatric patients for 7 consecutive days
Standard hospital inpatient menu (Full Ward Diet) for adult patients for 7 consecutive days
Vegetarian diet menu for paediatric for 7 consecutive days
Vegetarian diet menu for adult patients for 7 consecutive days
Short order menu food and drinks for paediatric and adult patients available in the 7 consecutive days
Oral nutrition support products for paediatric and adult patients available in the 7 consecutive days
**Exclusion**
Enteral, Parenteral, tube feedings
Therapeutically required menus (e.g., soft, liquid, texture modified, celiac diets)
Supplements
Foods purchased through vending machines or retail food outlets located in the hospitals are not part of the inpatient menu

3.2 Description of data to be collected

Detailed forms are presented at the end of this document.

- General information:
  - Foodservice model: room service; traditional pre-ordering; other
  - Meal preparation model: cook fresh only; predominantly cook fresh
  - Number of meals served per day

- Inpatient menus
  - Menu type: full ward diet for children, full ward diet for adults, vegetarian diet for children, and vegetarian diet for adults;
  - Meal category (breakfast, morning tea, lunch, afternoon tea, dinner, supper);

- Time of the meal service;
- Foods and meals description,
- Number of serves or amount (in grams or millilitres);
- Recipe of dishes prepared onsite, and respective serves/amounts;
- Product brand and name for packaged products;
- Identification of **<u>one</u>** ultra-processed food marker (see *3.2.1 Ultra-processed food markers*).

- List of all short order menu food and drinks:
  - Foods and drinks description;
  - Number of serves or amount (in grams or millilitres);
  - Recipe of dishes prepared onsite, and respective serves/amounts;
  - Product brand and name for packaged products;
  - Identification of **<u>one</u>** ultra-processed food marker (see *3.2.1 Ultra-processed food markers*).

- List of all oral nutrition support products:
  - Product brand and name for packaged products;
  - Number of serves or amount (in grams or millilitres);
  - Identification of **<u>one</u>** ultra-processed food marker (see *3.2.1 Ultra-processed food markers*).

3.2.1 Ultra-processed food markers

Ultra-processed food markers are identified based on Monteiro et al. *Ultra-processed foods: what they are and how to identify them. Public Health Nutr 2019;22(5):936-41. doi: 10.1017/S1368980018003762.*

Food substances not used in kitchens	
**Varieties of sugar**	• Fructose,<br>• High fructose corn syrup<br>• 'Fruit juice concentrates'<br>• Invert sugar<br>• Maltodextrin<br>• Dextrose, Dextrin<br>• Lactose<br>• Glucose syrup
**Modified oils**	• Hydrogenated<br>• Interesterified oils<br>• Margarine
**Protein sources**	• Hydrolysed proteins<br>• Soy protein isolate<br>• Gluten<br>• Casein<br>• Whey protein<br>• 'Mechanically separated meat'
**Other**	• Soluble or insoluble fibre<br>• Isolated fibre<br>• Modified starches

Cosmetic additives

Flavours *Can also be listed as:*

- Natural flavour
- Vanilla flavour
- Vanilla extract
- Aromas

Flavour enhancers *Can also be listed as:*

- MSG
- Monosodium/ Magnesium/ Monopotassium glutamate
- Maltol

Colours *Can also be listed as:*

- Natural colour
- Caramel (colour)
- Tartarzine
- Carmine

Emulsifiers *Can also be listed as:*

- Mono and diglycerides of fatty acids
- Polyglycerol polyricinoleate
- Soy lecithin
- Sunflower lecithin
- Diphosphates

Non-nutritive sweeteners *Can also be listed as:*

- Malitol
- Stevia leaf extract
- Steviol glycosides
- Steviol
- erythritol
- Acesulphame potassium
- Aspartame
- Sucralose
- Thaumatin
- Xylitol
- Neotame
- Mannitol

Thickeners *Can also be listed as:*

- Thickening agent
- Glycerine/glycerin/Glycerol
- Carrageenan

Gelling agents *Can also be listed as:*

- Gelatin
- Gums

Other

- Emulsifying salts
- Bulking agents
- Foaming agents
- Anti-foaming agents
- Carbonating agents
- Glazing agents

3.2.2 Examples

Example 1 – Impatient menu (breakfast)

**Date:** 22/09/2023	**Referring to day number: 1** *[Insert day 1 to 7]*				
**Menu type: Full ward diet for adults**					
[Insert menu type: Full Ward diet or vegetarian; adult/paediatric]					
**Please list all available options for each meal**					
**Food, drinks, and meals description**	**Serves/amount (g or ml)**	**Recipe**	**Serves/amount (g or ml) recipe**	**Packaged product brand and description**	**UPF marker***
**Breakfast - Time of the meal service: 07:00-8:30**					
Option 1:					
Cereal	2 serves			Uncle Tobys Plus Immune Support Breakfast Cereal	Colour
Full cream milk	2 x 150 ml			A2	None
OR option 2:					
Toast	2 pieces			Helga's Grain Bread Mixed Grain	Emulsifier
Margarine	2x			Essentials Table Spread Soft And Spreadable	Fully Hydrogenated Palm Oil
Spread	2x			Bega Peanut Butter Smooth	None
AND					
Yoghurt	1 serve			Yoplait Strawberry Yoghurt	Flavour
AND					
Fruit juice	1 serve			Original Juice Orange Juice	None

Example 2 - Short order menu food and drinks

**Date:** __/__/2023				**7-day reference period:** __/__/2023 to __/__/2023	
**Please list all short order menu food and drinks available in the 7 consecutive days of data collection**					
**Food and drinks description**	**Serves/amount (g or ml)**	**Recipe**	**Serves/amount (g or ml) recipe**	**Packaged product brand and description**	**UPF marker***
Lemonade	250 ml	Fresh lemon juice	3 tablespoons	Fresh	None
		Water	3/4 cup	Fresh	None
		White sugar	1 tablespoon	Csr White Sugar	None

Example 3 – Oral nutrition support

**Date:** __/__/2023	**7-day reference period:** __/__/2023 to __/__/2023	
**Please list all oral nutrition support products available in the 7 consecutive days of data collection**		
**Product brand and name**	**Serves/amount (g or ml)**	**UPF marker***
Nestle Resource 2.0 Vanilla Prism	237ml	Soy protein

3.3 Data collection procedures

Please complete at least one:

- Standard hospital inpatient menu (Full Ward Diet) for a group of patients (adults OR children) for 7 consecutive days
- Short order menu food and drinks for a group of patients (adults OR children) available in the 7 consecutive days
- Oral nutrition support products for a group of patients (adults OR children) available in the 7 consecutive days

Obs: If select 'adults' as the group, complete the inpatient menu, short order and oral nutrition support available for adults.

If time allows, please complete:

- Standard hospital inpatient menu (Full Ward Diet) for another group of patients (adults OR children) for 7 consecutive days
- Short order menu food and drinks for another group of patients (adults OR children) available in the 7 consecutive days
- Oral nutrition support products for another group of patients (adults OR children) available in the 7 consecutive days

- Vegetarian diet menu for adults and/or paediatric patients for 7 consecutive days

Forms for data collection are provided in Appendix 1. Hard copy forms will be entered into MicrosoftExcel (Microsoft Corporation, Redmond, WA) (spreadsheets created by the research team and shared within this e-mail).

Both scanning of hard copy forms and spreadsheets need to be sent to Priscila Machado via a password protected *Syncplicity* folder created specifically for this project. Only the researchers listed on this application will have access to the Syncplicity folder. You will only upload and see your own data in Syncplicity.

Please destroy all hard copies of collected forms and spreadsheets after sending to Priscila Machado (by 30th November 2023).

4. Additional information

Thank you very much for contributing to this research. You will be notified of the outcomes of this research via email and a copy of the publication(s) will be provided.

Supplementary material 2.

Data collection forms

Contribution of ultra-processed foods in a sample of Australian hospitals

Hospital: __________

Date: __/__/2023

Data collection form – general questions

1. What is the hospital's foodservice model?

☐ Room service

☐ Traditional pre-ordering

☐ Other: ____________________

2. What is the hospital's meal preparation model?

☐ Cook fresh only

☐ Predominantly cook fresh

3. Number of meals served in the hospital per day: ___________________

Contribution of ultra-processed foods in a sample of Australian hospitals

Hospital: __________

Data collection form – inpatient hospital menus

Date: __/__/2023 **Referring to day number:** ___ *[Insert day 1 to 7]*

Menu type: ____

[Insert menu type: Full Ward diet or vegetarian; adult/paediatric]

Please list all available options for each meal

Food, drinks, and meals description	Serves/amount (g or ml)	Recipe	Serves/amount (g or ml) recipe	Packaged product brand and description	UPF marker*
**Breakfast - Time of the meal service:** __________					
**Morning tea - Time of the meal service:** __________					

*UPF marker: Ultra-processed food marker, includes food substances not used in kitchens, e.g., hydrolysed proteins, soya protein isolate, gluten, casein, whey protein, mechanically separated meat', fructose, high-fructose corn syrup, 'fruit juice concentrate', invert sugar, maltodextrin, dextrose, lactose, soluble or insoluble fibre, hydrogenated or interesterified oil; and/or cosmetic additives such as flavours, flavour enhancers, colours, emulsifiers, emulsifying salts, sweeteners, thickeners, and anti-foaming, bulking, carbonating, foaming, gelling and glazing agents.

Food, drinks, and meals description	Serves/amount (g or ml)	Recipe	Serves/amount (g or ml) recipe	Packaged product brand and description	UPF marker*
**Lunch - Time of the meal service: __________**					
**Afternoon tea - Time of the meal service: __________**					
**Dinner - Time of the meal service: __________**					
**Supper - Time of the meal service: __________**					

*UPF marker: Ultra-processed food marker, includes food substances not used in kitchens, e.g., hydrolysed proteins, soya protein isolate, gluten, casein, whey protein, mechanically separated meat', fructose, high-fructose corn syrup, 'fruit juice concentrate', invert sugar, maltodextrin, dextrose, lactose, soluble or insoluble fibre, hydrogenated or interesterified oil; and/or cosmetic additives such as flavours, flavour enhancers, colours, emulsifiers, emulsifying salts, sweeteners, thickeners, and anti-foaming, bulking, carbonating, foaming, gelling and glazing agents.

Contribution of ultra-processed foods in a sample of Australian hospitals

Hospital: ___________

Data collection form – short order menu food and drinks

Date: __/__/2023 **7-day reference period:** __/__/2023 to __/__/2023

Please list all short order menu food and drinks available in the 7 consecutive days of data collection

Food and drinks description	Serves/amount (g or ml)	Recipe	Serves/amount (g or ml) recipe	Packaged product brand and description	UPF marker*

*UPF marker: Ultra-processed food marker, includes food substances not used in kitchens, e.g., hydrolysed proteins, soya protein isolate, gluten, casein, whey protein, mechanically separated meat', fructose, high-fructose corn syrup, 'fruit juice concentrate', invert sugar, maltodextrin, dextrose, lactose, soluble or insoluble fibre, hydrogenated or interesterified oil; and/or cosmetic additives such as flavours, flavour enhancers, colours, emulsifiers, emulsifying salts, sweeteners, thickeners, and anti-foaming, bulking, carbonating, foaming, gelling and glazing agents.

Contribution of ultra-processed foods in a sample of Australian hospitals

DEAKIN UNIVERSITY

Hospital: ___________

Data collection form – oral nutrition support products

Date: __/__/2023 **7-day reference period:** __/__/2023 to __/__/2023

Please list all oral nutrition support products available in the 7 consecutive days of data collection

Product brand and name	Serves/amount (g or ml)	UPF marker*

*UPF marker: Ultra-processed food marker, includes food substances not used in kitchens, e.g., hydrolysed proteins, soya protein isolate, gluten, casein, whey protein, mechanically separated meat', fructose, high-fructose corn syrup, 'fruit juice concentrate', invert sugar, maltodextrin, dextrose, lactose, soluble or insoluble fibre, hydrogenated or interesterified oil; and/or cosmetic additives such as flavours, flavour enhancers, colours, emulsifiers, emulsifying salts, sweeteners, thickeners, and anti-foaming, bulking, carbonating, foaming, gelling and glazing agents.

Supplementary material 3. STROBE checklist for cross-sectional studies

STROBE Statement—Checklist of items that should be included in reports of ***cross-sectional studies***

	Item No	Recommendation	Page No
**Title and abstract**	1	(*a*) Indicate the study's design with a commonly used term in the title or the abstract	1
		(*b*) Provide in the abstract an informative and balanced summary of what was done and what was found	1-2
**Introduction**			
Background/rationale	2	Explain the scientific background and rationale for the investigation being reported	3-4
Objectives	3	State specific objectives, including any prespecified hypotheses	4-5
**Methods**			
Study design	4	Present key elements of study design early in the paper	5
Setting	5	Describe the setting, locations, and relevant dates, including periods of recruitment, exposure, follow-up, and data collection	5
Participants	6	(*a*) Give the eligibility criteria, and the sources and methods of selection of participants	5
Variables	7	Clearly define all outcomes, exposures, predictors, potential confounders, and effect modifiers. Give diagnostic criteria, if applicable	5-6
Data sources/ measurement	8*	For each variable of interest, give sources of data and details of methods of assessment (measurement). Describe comparability of assessment methods if there is more than one group	5-6
Bias	9	Describe any efforts to address potential sources of bias	5-7
Study size	10	Explain how the study size was arrived at	5
Quantitative variables	11	Explain how quantitative variables were handled in the analyses. If applicable, describe which groupings were chosen and why	6-7
Statistical methods	12	(*a*) Describe all statistical methods, including those used to control for confounding	7
		(*b*) Describe any methods used to examine subgroups and interactions	n/a
		(*c*) Explain how missing data were addressed	n/a
		(*d*) If applicable, describe analytical methods taking account of sampling strategy	n/a
		(*e*) Describe any sensitivity analyses	n/a
**Results**			
Participants	13*	(a) Report numbers of individuals at each stage of study—eg numbers potentially eligible, examined for eligibility, confirmed	7

		eligible, included in the study, completing follow-up, and analysed	
		(b) Give reasons for non-participation at each stage	7
		(c) Consider use of a flow diagram	-
Descriptive data	14*	(a) Give characteristics of study participants (eg demographic, clinical, social) and information on exposures and potential confounders	7
		(b) Indicate number of participants with missing data for each variable of interest	n/a
Outcome data	15*	Report numbers of outcome events or summary measures	7
Main results	16	(*a*) Give unadjusted estimates and, if applicable, confounder-adjusted estimates and their precision (eg, 95% confidence interval). Make clear which confounders were adjusted for and why they were included	7-9
		(*b*) Report category boundaries when continuous variables were categorized	n/a
		(*c*) If relevant, consider translating estimates of relative risk into absolute risk for a meaningful time period	n/a
Other analyses	17	Report other analyses done—eg analyses of subgroups and interactions, and sensitivity analyses	n/a
**Discussion**			
Key results	18	Summarise key results with reference to study objectives	9-10
Limitations	19	Discuss limitations of the study, taking into account sources of potential bias or imprecision. Discuss both direction and magnitude of any potential bias	13
Interpretation	20	Give a cautious overall interpretation of results considering objectives, limitations, multiplicity of analyses, results from similar studies, and other relevant evidence	10-13
Generalisability	21	Discuss the generalisability (external validity) of the study results	12-13
**Other information**			
Funding	22	Give the source of funding and the role of the funders for the present study and, if applicable, for the original study on which the present article is based	Title page

*Give information separately for exposed and unexposed groups.

Note: An Explanation and Elaboration article discusses each checklist item and gives methodological background and published examples of transparent reporting. The STROBE checklist is best used in conjunction with this article (freely available on the Web sites of PLoS Medicine at http://www.plosmedicine.org/, Annals of Internal Medicine at http://www.annals.org/, and Epidemiology at http://www.epidem.com/). Information on the STROBE Initiative is available at www.strobe-statement.org.
